# Transcriptomics and metabolomics analysis reveal the anti-oxidation and immune boosting effects of mulberry leaves in growing mutton sheep

**DOI:** 10.3389/fimmu.2022.1088850

**Published:** 2023-02-27

**Authors:** Xiaopeng Cui, Yuxin Yang, Minjuan Zhang, Shuang Liu, Hexin Wang, Feng Jiao, Lijun Bao, Ziwei Lin, Xinlan Wei, Wei Qian, Xiang Shi, Chao Su, Yonghua Qian

**Affiliations:** College of Animal Science and Technology, Northwest A&F University, Yangling, Shaanxi, China

**Keywords:** mulberry leaves, anti-oxidation, peroxidation of polyunsaturated fatty acids, tryptophan metabolism, immunity, glycolysis

## Abstract

**Introduction:**

Currently, the anti-oxidation of active ingredients in mulberry leaves (MLs) and their forage utilization is receiving increasing attention. Here, we propose that MLs supplementation improves oxidative resistance and immunity.

**Methods:**

We conducted a trial including three groups of growing mutton sheep, each receiving fermented mulberry leaves (FMLs) feeding, dried mulberry leaves (DMLs) feeding or normal control feeding without MLs.

**Results:**

Transcriptomic and metabolomic analyses revealed that promoting anti-oxidation and enhancing disease resistance of MLs is attributed to improved tryptophan metabolic pathways and reduced peroxidation of polyunsaturated fatty acids (PUFAs). Furthermore, immunity was markedly increased after FMLs treatment by regulating glycolysis and mannose-6-phosphate pathways. Additionally, there was better average daily gain in the MLs treatment groups.

**Conclusion:**

These findings provide new insights for understanding the beneficial effects of MLs in animal husbandry and provide a theoretical support for extensive application of MLs in improving nutrition and health care values.

## Introduction

Currently, a number of medicinal plants are widely used as functional foods and alternative medicine to prevent and treat chronic diseases (1). This is due to the numerous bioactive components with anti-oxidant capabilities, such as phenolic compounds and flavonoids (2), which may help improve immunity by coordinating the metabolism of the body. Mulberry leaves (MLs) have been used as feed for silk worms for hundreds of years and also as a traditional Chinese medicine, according to the classical medicine books. Owing to their anti-oxidative, anti-inflammatory, anti-bacterial, and anti-hyperlipidemic properties, mulberry leaves are gaining increasing attention for use in Chinese herbal medicines (3). To date, the hypoglycemic and lipid-lowering effects of extracts or active ingredients in MLs are well established, against diabetes, fatty liver, and some similar diseases related to disorders in glucose and lipid metabolism. In addition, accumulating evidence have validated the promotion of growth and rumen development, anti-oxidant properties, and improvement in milk production by MLs or their active ingredients in livestock (3–6). However, the underlying mechanism of MLs, as an unconventional feed with both nutritive and medicinal properties, on anti-oxidation and immunity in livestock remains poorly understood. In recent years, an explosion has occurred in the acquisition of biological data through the use of so-called ‘omics’ techniques. Whilst many different omics technologies are now featured in the literature, the most frequently used omics are genomics, transcriptomics, proteomics and metabolomics (7). Thus, the aim of the present study was to evaluate the roles of MLs in growth promoting and animal welfare improving aspects of mutton sheep in terms of antioxidant and immune properties and explore the mechanism by methods of transcriptomic and metabolomic.Our study provides novel insights into the role of MLs in livestock yield and the application of natural functional fodder.

## Materials and methods

The experiment was conducted in accordance with the Chinese Guidelines for Animal Welfare and Experimental Protocols, and approved by the Animal Care and Use Committee of the Institute of Northwest A & F University.

### Preparation and chemical indexes measurement of fermented mulberry leaves and dried mulberry leaves

MLs (species 707) are harvested in July 2021 at the Institute of Sericulture and Silk in Zhouzhi, Shaanxi Province, China. One half is sun-dried for seven days, next well-sealed in woven bags after a little rubbing and then stored in a dry, dark place for acquiring DMLs for use in feeding experiment. The other half with 65.03% moisture content after wilted by sun-shine for a half day, is a little smashed and vacuum sealed in fermentation-special bags to ferment with 5% *Lactobacillus plantarum* inoculation at room temperature (27.5-28°C) for thirty days in a dry, dark place for preparation for FMLs. Here 5% is adding 5 mL of bacterial culture suspension to 100 grams of MLs and the concentration of bacterial culture suspension is 1×10^8^ CFU/mL. *Lactobacillus plantarum* (CICC 23941) purchased from the China Center of Industrial Culture Collection (www.china-cicc.org). Before feeding experiments, the pH, crude protein, crude fiber as well as gross energy of FMLs and DMLs are determined according to standard methods of AOAC. And their contents are shown in **Table 1**. FMLs are deemed qualified without aflatoxin B1 detected at a minimum checked value of 0.1 μg/kg by Huayan Testing Group Co., Ltd in Xi’an City, Shaanxi Province (Detection number: SP202115450).

**Table 1:** Chemical composition of FMLs and DMLs.

Items	DM loss/%	pH	Crude protein/%	Crude fiber/%	Gross energy/(MJ/kg)
FMLs	4.6	3.98	14.69	8.35	15.92
DMLs		6.20	15.15	9.72	14.28

FMLs, fermented mulberry leaves; DMLs, dried mulberry leaves.

### Experimental design and feeding diets

Animal experiments are conducted on six-month-old healthy female mutton sheep (white-headed Suffolk sheep♂×Hu sheep♀) weighing 30.41kg at average without genetic modification in Gansu Qinghuan Meat Sheep Seed Production Co. Ltd (Huan County, Qingyang City, Gansu Province, China). The animals were randomly assigned to group Con feeding a normal control diet (n=18), group TR1 feeding an experimental diet with FMLs (n=18) and group TR2 feeding an experimental diet with DMLs (n=18) and then treated for an experiment of fifty days. Each group had 6 replicates with 3 sheep per replicate. Before the feeding experiment, animals undergo an acclimatization period of six days to obtain an appropriate feed intake, during which they were allowed unlimited access to their corresponding experimental diet and tap water. Experimental sheep were housed in sheepfold and given self-help feeding in three groups every day. The ingredients and chemical composition of three experimental diets are shown in **Table 2**. The chemical compositions of three experimental diets are determined by Ulanqab Yima Agriculture and Animal Husbandry Technology Co., Ltd (Ulanqab City, Inner Mongolia, China).

**Table 2:** Ingredients and chemical composition of three experimental diets.

Items	Con	TR1	TR2
Ingredients			
DMLs/%	0	0	7.11
FMLs/%	0	16.59	0
Oat hay/%	12.43	24.88	17.77
Corn silage/%	29.00	8.29	24.88
Corn/%	19.34	16.59	16.59
Wheat/%	20.72	17.77	17.77
Concentrate/%	17.68	15.17	15.17
Limestone/%	0.83	0.71	0.71
Total/%	100	100	100
Nutrients (based on dry matter)			
Dry matter/%	72.50	66.10	66.40
Crude protein/%	16.40	16.70	16.60
Metabolizable Energy/(MJ/kg)	10.46	10.63	10.30
Crude fat/%	3.00	3.40	3.00
Crude ash/%	9.58	9.24	10.59
Acid detergent fiber/%	16.80	17.50	15.30
Neutral detergent fiber/%	27.80	28.47	25.76
Lignin/%	4.30	4.40	4.80

### Weighing and sample collection

Prior to the experiments, all sheep are driven to be weighed by an automatic weighing system to obtain the initial body weights. Afterwards, body weights on day 25th and 50th are weighted to calculate daily gains. On the 50th day, blood from the jugular vein was collected and placed in 5mL vacuum negative-pressure tubes with yellow cap containing separation gels for serum separation and then leave to set for one to two hours to collect rough 2.5mL serum, which is immediately stored in liquid nitrogen and taken to the lab for further analysis. At the end of the experimental period, 6 sheep per group which were representative in terms of average weight (inclusion criteria) of group were selected and slaughtered for tissue sample collection. Tissue samples (about 0.5×0.5×0.5cm^3^) of longissimus dorsi muscle and subcutaneous fat from the left side of the carcass are packed into 2 mL cryopreserved tube and frozen in liquid nitrogen immediately within 20 min of slaughter for biochemical indexes and omics analysis. Weighting samples contain 18 biological repeats of each group.

### Analysis of biochemical indexes

Growth hormone (GH), total antioxidant capacity (TAOC), superoxide dismutase (SOD), catalase (CAT), glutathione peroxidase (GSH-Px) and malondialdehyde (MDA) are determined using commercially available kits (HY-60021, HY-60001, HY-M0018, HY-60005, HY-60003; Beijing Huaying Biotechnology Research Institute, Beijing, China). Serum immunoglobulin A, M, G (IgA, IgM, IgG) and tumor necrosis factor (TNFα) were determined by commercially available kits (HY-N0048, HY-N0049, HY-N0050, HY-H0019; Beijing Huaying Institute of Biotechnology Research Institute, Beijing, China). Immunoglobulin (IG) is the sum of immunoglobulins IgA, IgM and IgG. Muscle tissue samples contain 5 biological repeats (group Con), 6 biological repeats (group TR2) and 5 biological repeats (group TR2), respectively. Adipose tissue samples contain 5 biological repeats (group Con), 5 biological repeats (group TR2) and 5 biological repeats (group TR2), respectively. Serum samples contain 8 biological repeats (group Con), 7 biological repeats (group TR2) and 8 biological repeats (group TR2), respectively. Technical repetition is no less than 2 for all samples. No data point from the analysis is excluded.

### Widely target metabolomics analysis

Tissue samples of muscle are extracted by Metware according to standard procedures. The sample extracts were analyzed using an LC-ESI-MS/MS system (UPLC, ExionLC AD, https://sciex.com.cn/; MS, QTRAP^®^ System, https://sciex.com/). LIT and triple quadrupole (QQQ) scans were acquired on a triple quadrupole-linear ion trap mass spectrometer (QTRAP), QTRAP^®^ LC-MS/MS System, equipped with an ESI Turbo Ion-Spray interface, operating in positive and negative ion mode and controlled by Analyst 1.6.3 software (Sciex). Instrument tuning and mass calibration were performed with 10 and 100 μmol/L polypropylene glycol solutions in QQQ and LIT modes, respectively. A specific set of MRM transitions were monitored for each period according to the metabolites eluted within this period. Significantly regulated metabolites between groups were determined by variable importance in projection (VIP)≥1 and absolute Log_2_FC (fold change)≥1. VIP values were extracted from OPLS-DA result, which also contain score plots and permutation plots, was generated using R package MetaboAnalystR. The data was log transform (log_2_) and mean centering before OPLS-DA. In order to avoid overfitting, a permutation test (200 permutations) was performed.

### Transcriptomic analysis

RNA-extract and RNA-seq of muscle are conducted according to standard procedures of Majorbio with the Illumina HiSeq xten/NovaSeq 6000 sequencer (2×150bp read length). The raw paired end reads were trimmed and quality controlled by SeqPrep (https://github.com/jstjohn/SeqPrep) and Sickle (https://github.com/najoshi/sickle) with default parameters. Then clean reads were separately aligned to reference genome with orientation mode using HISAT2 (http://ccb.jhu.edu/software/hisat2/index.shtml) software (8). The mapped reads of each sample were assembled by StringTie (https://ccb.jhu.edu/software/stringtie/index.shtml?t=example) in a reference-based approach (9). To identify DEGs (differential expression genes) between two different samples, the expression level of each transcript was calculated according to the transcripts per million reads (TPM) method. RSEM (http://deweylab.biostat.wisc.edu/rsem/) (10) was used to quantify gene abundances. Essentially, differential expression analysis was performed using the DESeq2 (11)/DEGseq (12)/EdgeR (13) with Q value ≤ 0.05, DEGs with |log2FC|>1 and Q value ≤ 0.05(DESeq2 or EdgeR)/Q value ≤ 0.001(DEGseq) were considered to be significantly different expressed genes. The transcriptomic sequence data have been deposited in the NCBI database (Accession No. PRJNA898816).

### Statistical analysis

Statistical analysis was performed by the SPSS 19.0 software (IBM-SPSS Statistics, IBM Corp., Armonk, NY, United States). Data were evaluated using a one-way ANOVA followed by Turkey’s multiple range tests for physiological and biochemical indexes. Significance was declared if *p*<0.05. Additionally, omics sequencing data are analyzed using online platforms for data analysis, including Metware cloud tools (https://cloud.metware.cn/#/tools/tool-list) and Majorbio cloud platform (https://cloud.majorbio.com/). Histograms and metabolic pathway maps are drawn respectively using Graphpad Prism 8 and Adobe Illustrator CS6.

## Results

### Growth performance

Throughout the trial, no significant differences were detected in daily gain (0–25d) (Con<TR2<TR1) and feed to gain ratio (F/G) (Con>TR2>TR1) (*p*>0.05, **Table 3**), although group TR1 which were fed with FMLs demonstrated a little increase in daily gain (0–25d) and a slight decrease in F/G. Apparently, treatments with FMLs and DMLs (group TR2) generated an obvious increase in ADFI during the overall raising period (*p*<0.05), which suggests MLs are a delicious feed for promotion. In addition, ADG, daily gain (25–50d) and serum growth hormone levels were significantly improved in both MLs-treatment groups (*p*<0.05) in the study. Further, FMLs feeding resulted in a significant increase in the final body weight (*p*<0.05).

**Table 3:** Growth performance.

Items	Con	TR1	TR2	SEM	*P*-value
Initial BW/kg	30.36	30.36	30.50	0.589	0.994
Final BW/kg	38.03^b^	42.05^a^	40.86^ab^	0.696	0.050
ADFI/kg	1.79^b^	2.31^a^	2.35^a^	0.033	0.000
Daily gain (0-25d)/g	95.56	125.56	97.64	6.999	0.145
Daily gain (25-50d)/g	211.11^b^	342.22^a^	317.64^a^	16.330	0.001
ADG (0-50d)/g	156.11^b^	233.89^a^	205.56^a^	7.876	0.001
F/G	13.38	10.26	11.79	1.026	0.483
GH	4.81^c^	5.84^b^	7.28^a^	0.265	0.000

BW, body weight; ADFI, average daily feed intake; ADG, average daily gain; F/G, ADFI/ADG; GH, growth hormone; SEM, standard error mean; Different letters in the same row (a–c) differed (p<0.05).

### Anti-oxidant properties

As shown in **Table 4**, SOD (superoxide dismutase), CAT (catalase), GSH-Px (glutathione peroxidase) and TAOC (total antioxidant activity) in serum and muscle were significantly increased in the MLs treatment group, especially in the FMLs treatment group (*p*<0.05); SOD and GSH-Px in adipose tissue also increased significantly (*p*<0.05), CAT and TAOC tended to increase (Con<TR2<TR1, *p*>0.05). In addition, feeding MLs significantly decreased the content of MDA in serum and muscle of mutton sheep (*p*<0.05), the content of MDA in adipose tissue was Con>TR2>TR1 (*p*>0.05).

**Table 4:** Anti-oxidant properties of serum, muscle and adipose tissues.

Items	Con	TR1	TR2	SEM	*P*-value
Serum					
SOD	58.72^c^	77.94^a^	66.56^b^	2.021	0.000
CAT	32.80^c^	58.95^a^	45.22^b^	2.489	0.000
GSH-PX	358.13^c^	543.05^a^	476.41^b^	17.737	0.000
TAOC	7.37^c^	10.84^a^	8.49^b^	0.366	0.000
MDA	5.09^a^	4.05^b^	4.60^ab^	0.150	0.013
Muscle					
SOD	5.55^c^	9.64^a^	7.67^b^	0.475	0.000
CAT	2.37^c^	4.49^a^	3.56^b^	0.241	0.000
GSH-PX	23.12^c^	31.81^a^	27.35^b^	1.029	0.000
TAOC	3.18^c^	5.10^a^	4.47^b^	0.226	0.000
MDA	4.33^a^	3.31^b^	3.98^a^	0.134	0.001
Adipose					
SOD	2.18^c^	5.23^a^	3.58^b^	0.371	0.000
CAT	0.72	1.68	0.98	0.201	0.126
GSH-PX	6.01^c^	11.17^a^	8.20^b^	0.691	0.003
TAOC	0.98	2.05	1.26	0.254	0.212
MDA	1.32	1.17	1.00	0.185	0.809

SOD, superoxide dismutase; CAT, catalase; GSH-PX, glutathione peroxidase; TAOC, total antioxidant capacity; MDA, malonaldehyde; muscle, longissimus dorsi, adipose, subcutaneous fat. Different letters in the same row (a–c) differed (p<0.05).

**Figure 1:**
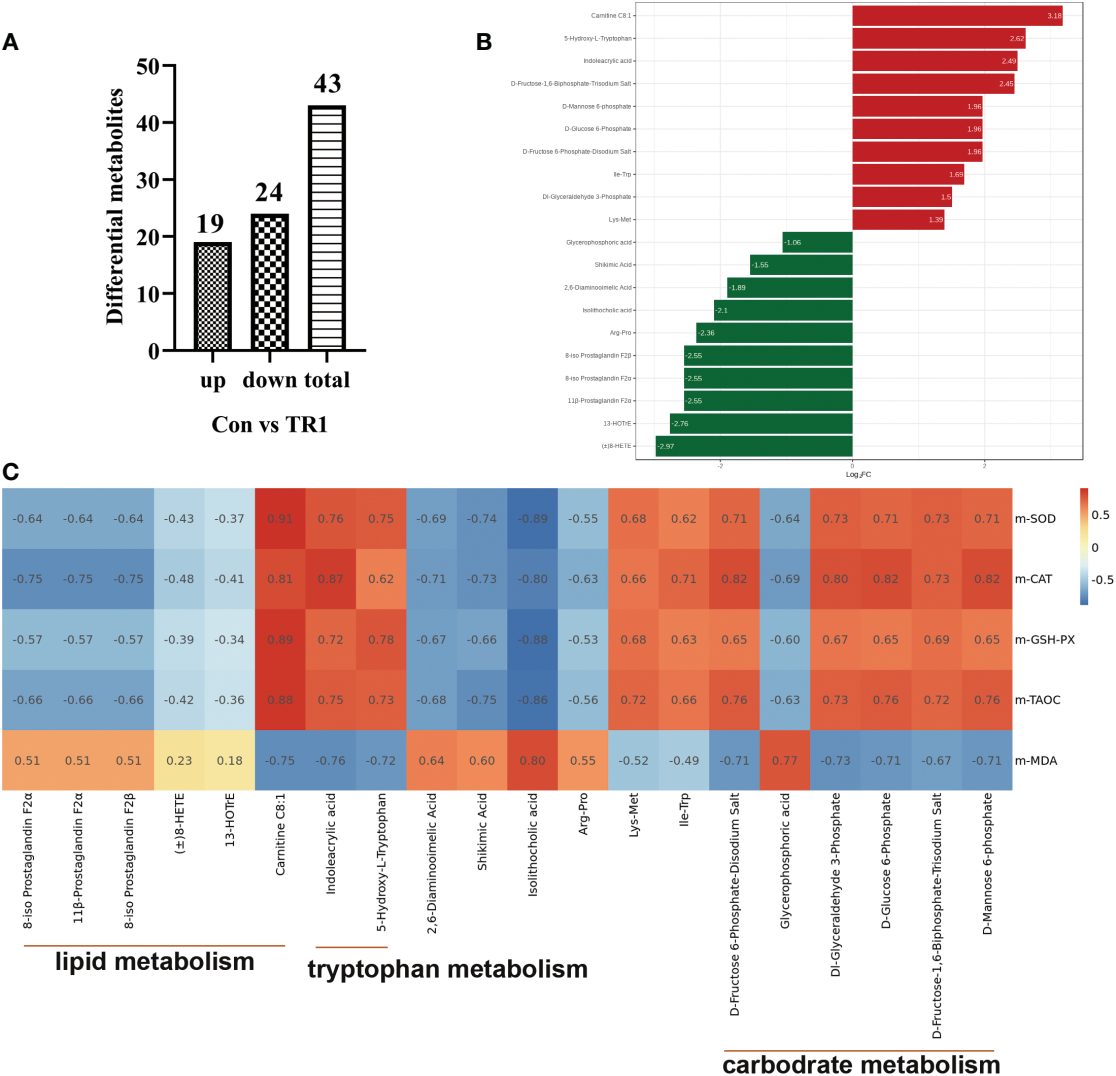
DEMs from Con vs. TR1. Upregulated, downregulated and total numbers of DEMs from Con vs. TR1 **(A)**, the 20 leading DEMs from Con vs. TR1 **(B)**, a correlation heat map between the 20 leading DEMs from Con vs. TR1 and their indexes of antioxidant performance **(C)**.

**Figure 2:**
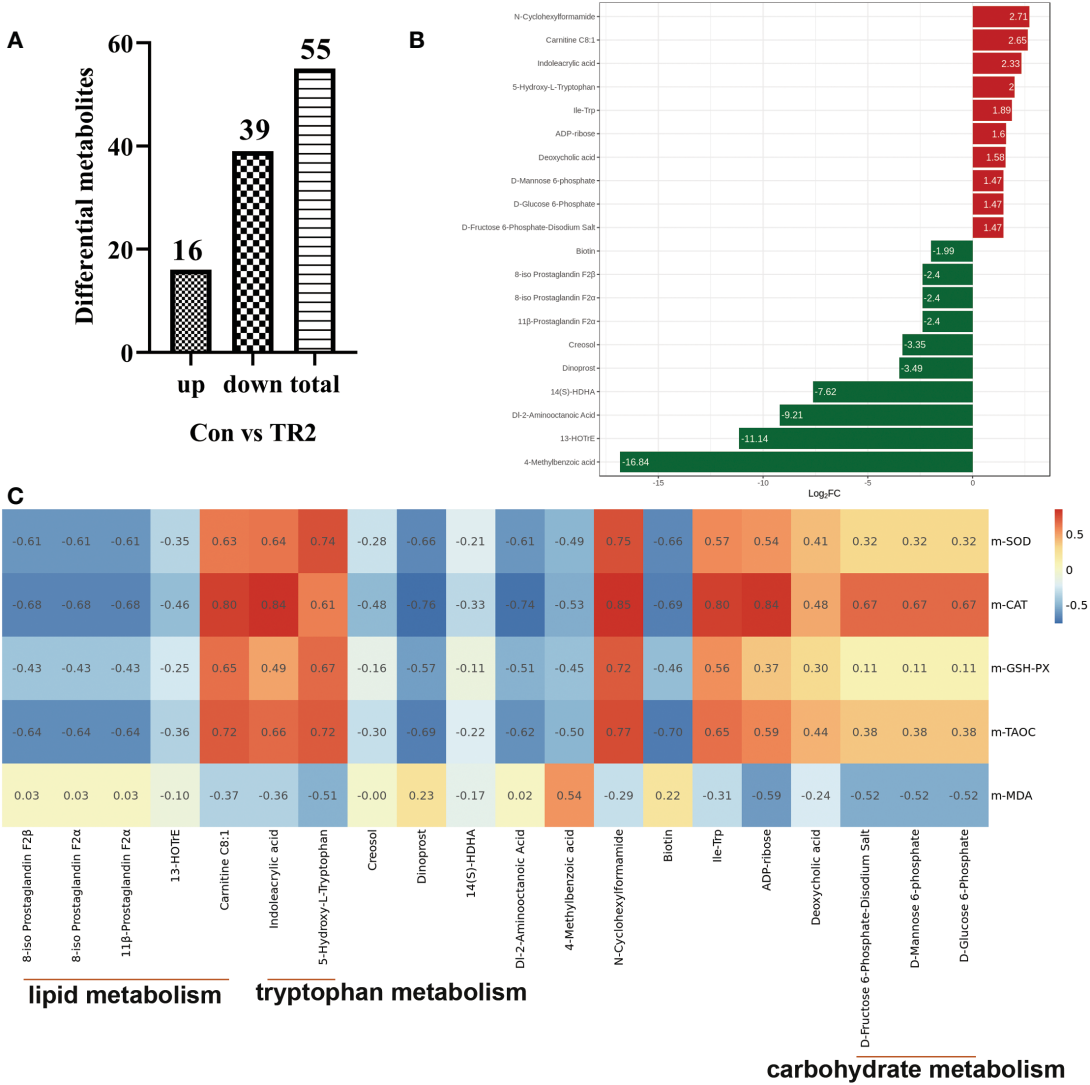
DEMs from Con vs. TR2. Upregulated, downregulated and total numbers of DEMs from Con vs. TR2 **(A)**, the 20 leading DEMs from Con vs. TR2 **(B)**, a correlation heat map between the 20 leading DEMs from Con vs. TR2 and their indexes of antioxidant performance **(C)**.

To further explore how MLs cause a differences in promoting oxidation resistance, muscle widely target metabolomics was applied. A total 43 significant differential metabolites (DEMs), including 19 upregulated and 24 downregulated DEMs after FMLs treatment, were filtered according to the criteria that the metabolite contents were within FC≥2 or FC ≤ 0.5, and VIP≥1 (**Figure 1A**). On this basis, the p-value is listed ascending order and absolute value of log_2_FC is listed in descending order of 43 DEMs to further obtain the leading 20 DEMs, shown in **Figure 1B**. These top-ranking DEMs were mainly involved in lipid, carbohydrate, amino acid, and organic acid metabolism. As the heatmap shows, anti-oxidant properties were negatively correlated (dark blue) with products of lipid metabolism (8-iso Prostaglandin F2α, 11β-Prostaglandin F2α, 8-iso Prostaglandin F2β, (±)8-HETE, 13-HOTrE, Carnitine C8:1), and were positively correlated (dark red) with D-Glucose 6-Phosphate, D-Fructose 6-Phosphate-Disodium Salt, D-Fructose-1,6-Biphosphate-Trisodium Salt, and D-Mannose 6-phosphate, which are related to carbohydrate metabolism (**Figure 1C**). This suggests that lipid metabolism and carbohydrate metabolism in FMLs treatment regulate the anti-oxidant process. More importantly, the correlation analysis suggests that the increased expression of 5-Hydroxy-L-Tryptophan and indoleacrylic acid produced by tryptophan metabolism may play crucial roles in anti-oxidant regulation. **Figure 2A** exhibited the DEMs from Con vs. TR2,including 16 upregulated and 39 downregulated, 55 in total DEMs, based on the same screening criteria as FMLs treatment. Similarly, the top-ranking 20 DEMs in **Figure 2B** obtained in the same method, are also mainly involved in lipid metabolism (8-iso Prostaglandin F2α, 11β-Prostaglandin F2α, 8-iso Prostaglandin F2β, 13-HOTrE, Carnitine C8:1), carbohydrate metabolism (D-Mannose 6-phosphate, D-Glucose 6-Phosphate, D-Fructose 6-Phosphate-Disodium Salt), amino acid metabolism and organic acid metabolism. However, few correlation relationships (yellow in **Figure 2C**) between carbohydrate metabolism and anti-oxidant properties in heatmap analysis show that DMLs supplementation might slightly, or not facilitate oxidation resistance by regulating carbohydrate metabolism.

**Figure 3:**
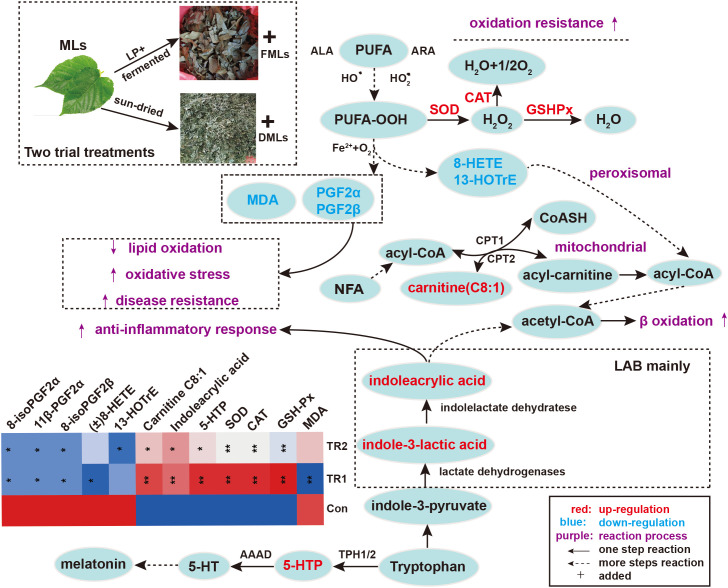
Peroxidation of PUFAs and tryptophan metabolism. Elevated metabolites are highlighted in red, reduced metabolites are shown in blue; the contents of painted green or red metabolites from top 20 DEMs and antioxidant biochemical indexes are displayed in heat map (*p<0.05, **p<0.01, * and ** are TR1 or TR2 compared to Con). (MLs, mulberry leaves; FMLs, fermented mulberry leaves; DMLs, dried mulberry leaves; LP, *Lactobacillus plantarum*; LAB, lactic acid bacteria; PUFA, polyunsaturated fatty acids; ALA, α linolenic acid; ARA, arachidonic Acid; CPT1/CPT2, carnitine palmitoyltransferase 1/2; NFA, medium-chain fatty acid; 5-HTP, 5-hydroxytryptophan; 5-HT, serotonin; AAAD, aromatic amino acid decarboxylase; TPH1/2, tryptophan hydroxylase 1/2).

Considering the 20 DEMs and antioxidant performance indexes between Con vs. TR1 and Con vs. TR2, It is not too difficult to discover the importance of lipid metabolism, especially the peroxidation of polyunsaturated fatty acids (PUFAs), amino acid metabolism (mainly tryptophan metabolism) for MLs treatment, and carbohydrate metabolism (mainly glycolysis and mannose 6-phosphate pathway) only for FMLs treatment in promoting oxidation resistance. The tryptophan metabolism and peroxidation of PUFAs could be promising MLs-dependent biomarkers of the anti-oxidant metabolism pathway. Indoleacrylic acid and 5-hydroxy tryptophan (5-HTP) obtained from the two routes of tryptophan metabolism (**Figure 3**) were significantly upregulated. Indolelactic acid, an upstream metabolite of indoleacrylic acid, was also significantly increased in FMLs treatment (*p*=0.015). Moreover, the markedly decreased 8-HETE, 13-HOTrE, 8-iso Prostaglandin F2α, 11β-Prostaglandin F2α, and 8-iso prostaglandin F2β levels and increased carnitine C8:1 are present after MLs treatment.

**Figure 4:**
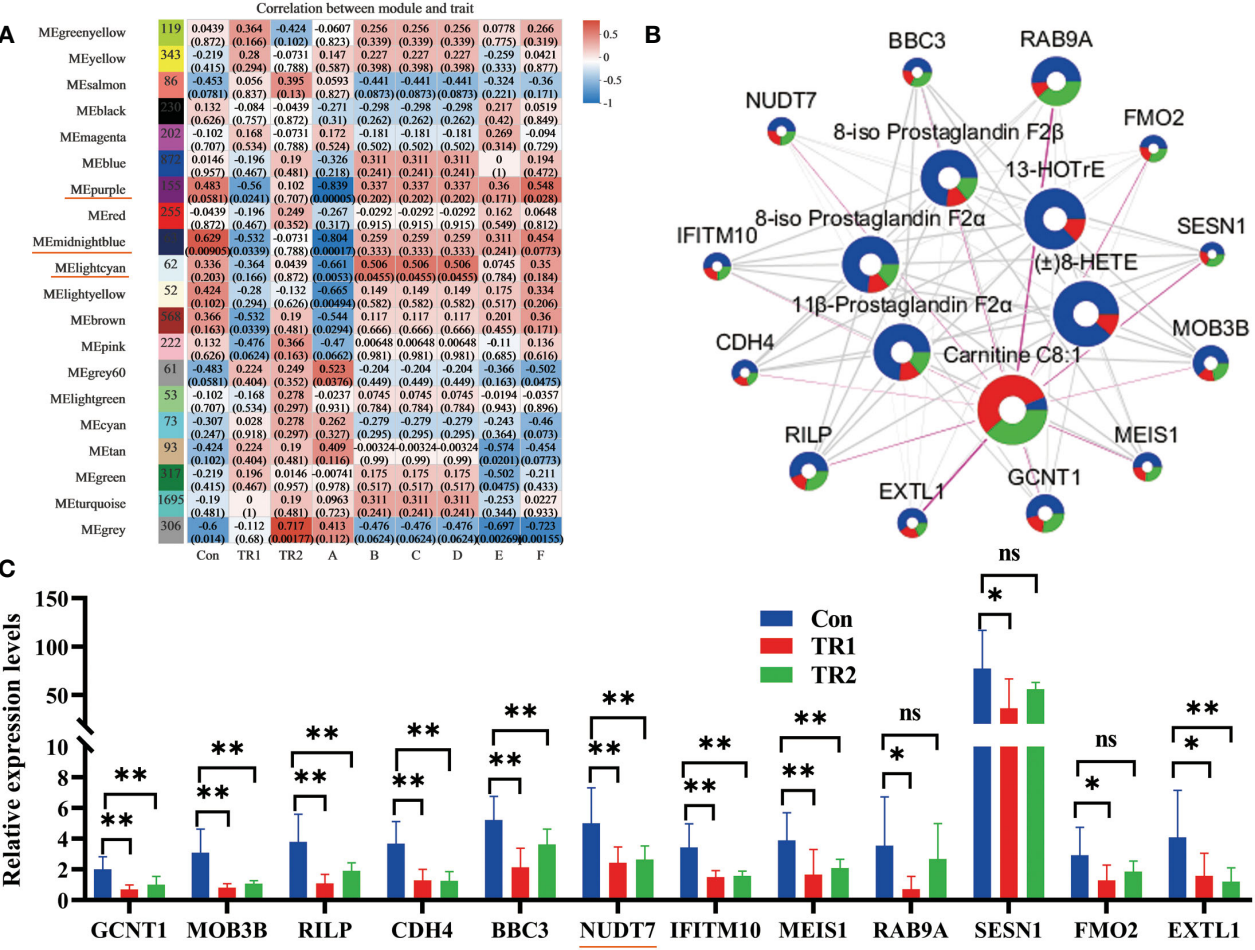
DEGs related with peroxidation of PUFAs. Module analysis of DEMs related with PUFAs metabolism and all filtered genes (Underlined modules in red represent selective modules; A, carnitine C8:1; B, 8-iso-prostaglandin F2α; C, 11β-prostaglandin F2α; D, 8-iso-prostaglandin F2β; E, (±) 8-HETE; F, 13-HOTrE) **(A)**, network map analysis of selective twelve DEGs and DEMs related with PUFAs metabolism (circle size represents absolute log_2_FC (Con vs TR1) value; blue, red and green divisions in every circle are on behalf of contents of some DEGs or DEMs in Con, TR1,TR2 in turn; The thickness of the connecting wire represents the degree of connectivity) **(B)** and the relative expression levels of selective twelve target DEGs in three groups (*represents *p*<0.05, ** represents *p*<0.01, ns represents no differences) **(C)**.

Transcriptome analysis was performed to further verify that reducing the peroxidation of PUFAs could indeed promote oxidation resistance. All filtered sequenced genes were used for weighted gene co-expression network analysis (WGCNA) analysis. Correlation analysis of different module genes and grouping factors and six DEMs related to PUFAs metabolism (8-iso Prostaglandin F2α, 11β-Prostaglandin F2α, 8-iso Prostaglandin F2β, (±)8-HETE, 13-HOTrE, carnitine C8:1) are shown in **Figure 4A**. Three module genes (underlined module in red in the **Figure 4A**) with almost the same correlation with the grouping factors and DEMs were integrated for further analysis. Subsequently, 14 target DEGs were obtained from the integrated genes with two criteria that their p-value must be less than 0.05, and absolute log_2_FC (Con vs. TR1) value must be not less than 1. Subsequently, they were gathered with six DEMs for network map analysis (**Figure 4B**). The relative expression levels of these 14 target DEGs in the three groups are shown in **Figure 4C**. Relative expression levels of *GCNT1*, *IFITM10*, *EXTL1*, *RILP*, *BBC3*, *RAB9A*, *MOB3B*, *SESN1*, *CDH4*, *MEIS1*, *RAB9A*, *NUDT7*, *FMO2* and *NUDT7* was decreased siginificantly (*p*<0.05).

### Immune response

**Figure 5:**
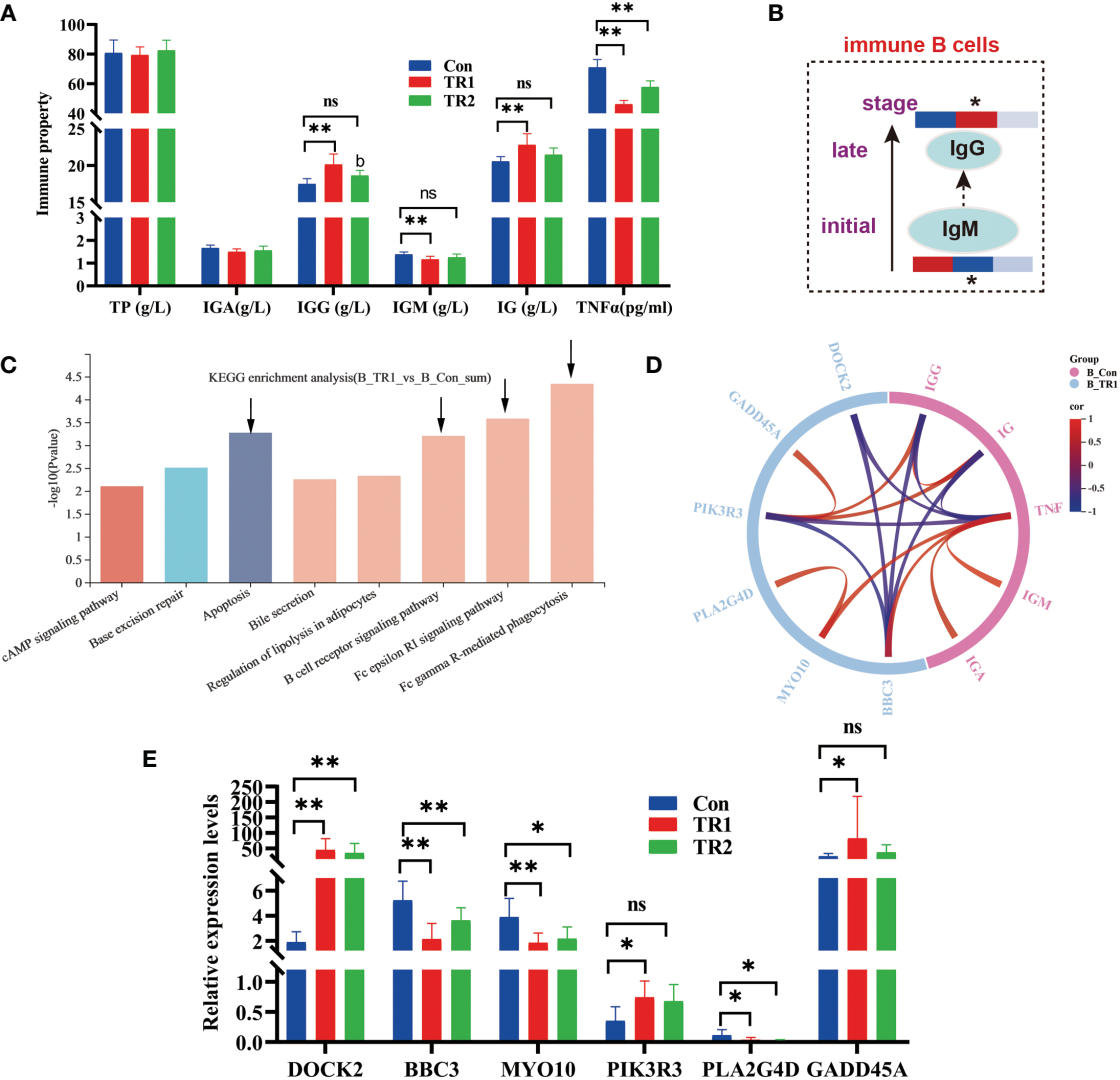
Indexes of immune properties and DEGs related to immune response. Immune indexes of serum **(A)** a transition from IgM to IgG in immune B cells over the course of immune response (Heat maps show the relative amounts of substances in group Con, TR1 and TR2 from left to right; * represent *p*<0.05, indicative of the significant difference by comparing TR1 or TR2 to Con) **(B)**, kegg enrichment analysis of all DEGs from TR1 vs. Con **(C)**, Circular correlation analysis of six selective DEGs from top 4 kegg pathway and immune indexes of serum **(D)**, Relative expression levels of six selective DEGs related to immune response **(E)** (* represents *p*<0.05, ** represents *p*<0.01, ns represents no differences in histograms).

Serum immuno globulin G (IgG) and total immuno globulin (Ig) levels increased in the FMLs (*p*<0.05) remarkably and DMLs fed groups (*p*>0.05). The pro-inflammatory tumor necrosis factor-α (TNF-α) was dramatically reduced by both MLs treatments (**Figure 5A**) (*p*<0.05). However there is a distinctive decrease in immuno globulin M (IgM) following FMLs treatment (*p*<0.05). The decreased IgM following FMLs treatment is related to a transition in antibody class from IgM to IgG, over the course of an immune response (14) (**Figure 5B**).

As reported by Wu et al. (15), muscles support a strong immune response. To validate the promotion of the immune process of FMLs, all DEGs of FMLs treatment in muscle analyzed by transcriptomics were applied for enrichment analysis, and the top eight KEGG pathways are represented in a histogram (**Figure 5C**). The four leading enriched pathways (arrow’s place in **Figure 5C**) are closely related to apoptosis and immune processes. Subsequently, a total of six annotated DEGs from the leading four pathways and immune indices were combined to analyze the relevance and a clear relationship was shown in the circular map (**Figure 5D**). After MLs treatment, the relative expressions of the DEGs, including *DOCK2*, *BBC3*, *MYO10, PIK3R3, PLA2G4D*, *GADD45A*, were markedly altered (**Figure 5E**).

**Figure 6:**
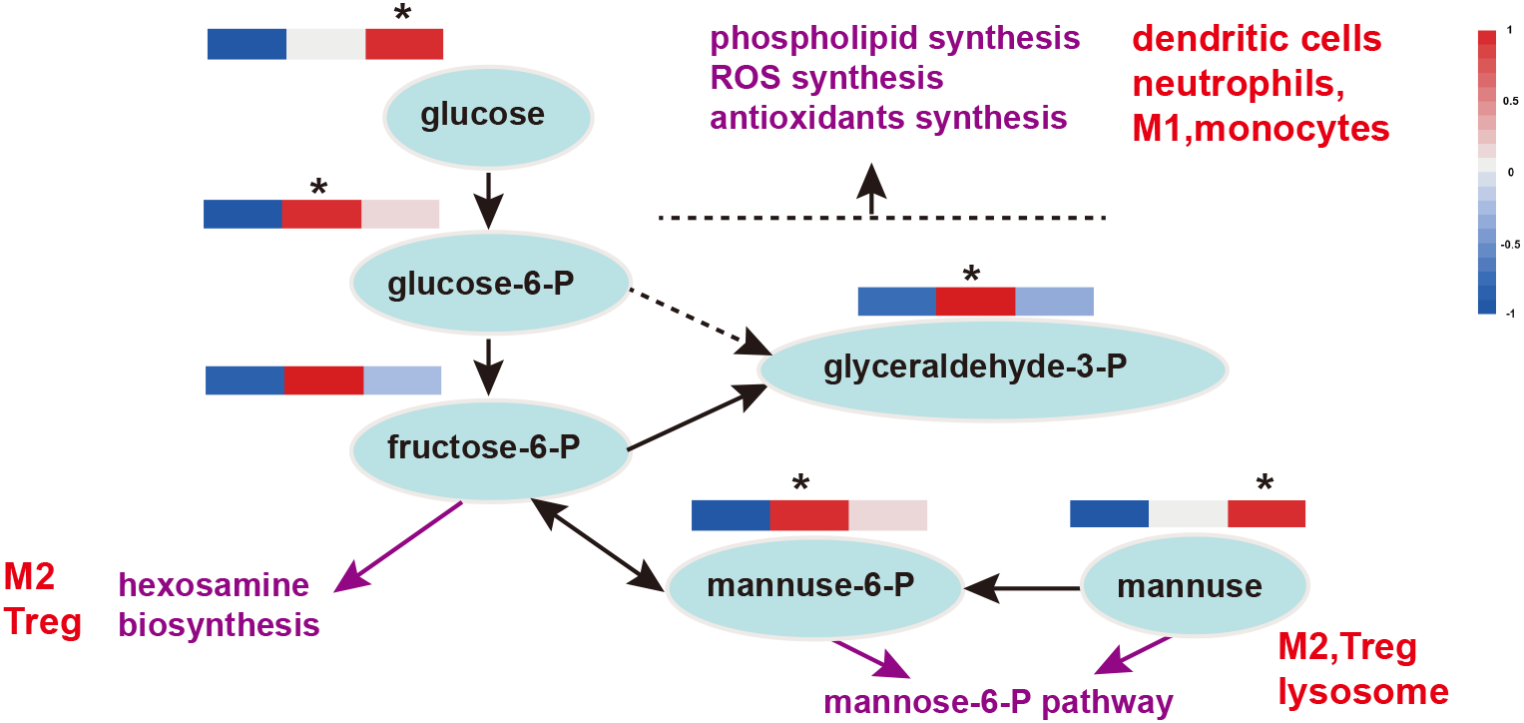
Glycolysis and the mannose-6-P pathway in immune function. Heat maps show the relative amounts of substances in group Con, TR1 and TR2 from left to right; * represent *p*<0.05, indicative of the significant difference by comparing TR1 or TR2 to Con; M1, type 1 macrophages; M2, type 2 macrophages; Treg, regulatory T cells; P, phosphatase.

Previously, we found that FMLs improves carbohydrate metabolism, and glycolysis is one of the key processes. It has been found that it can provide biosynthetic intermediates and reducing power for the growth and proliferation of immune cells. MLs treatments raise levels of glucose, the central substrate of glycolysis and FMLs supplementation significantly increases the contents of glucose-6-P, glyceraldehyde-3-P (*p*<0.05) and almost significantly increases fructose-6-P (*p*=0.054) (**Figure 6**). Additionally, there are significantly increased D-mannose in DMLs treatment (*p*<0.05) and mannose-6-P in FMLs treatment (*p*<0.05) (**Figure 6**). These two are both derived from mannose-6-P pathway.

## Discussion

Numerous studies have shown the diverse growth-promoting effects of MLs (4, 16). Our study also proves this point. In addition, this study also found that FMLs are superior to FMLs in palatability and growth promotion, which is a rare feed additive, and its application prospects in animal husbandry production appear considerable.

### Anti-oxidation activity

Oxidation in biological systems is mainly mediated by a series of redox enzymes. Peroxidation caused by free radical chain reactions may lead to oxidative stress (17). SOD, CAT and GSH-Px are common enzymatic antioxidants. SOD can convert free radicals (O2−•) generated in the body’s peroxidation reaction into H2O2 (18), and H_2_O_2_ can then be converted into H_2_O by CAT and GSH-Px to reduce the damage resulting from free radical to the body and improve antioxidant performance. MDA is one of the representative end products under non-enzymatic lipid peroxidation, indicating the extent of lipid peroxidation (19). Meanwhile, MDA is also an important indicator of membrane damage and body aging, and one of the toxic substances produced by the increase of ROS (20). The increase of SOD, CAT, GSH-Px, TAOC and the decrease of MDA in this study all indicate that MLs can improve the antioxidant performance of the body, which is consistent with the results of previous studies (3, 21–23). The reason is that MLs are rich in bioactive ingredients. In addition, this study also found that FMLs have the strongest antioxidant properties, mainly because of their higher active ingredients than DMLs (24).

Indoleacrylic acid derived from tryptophan metabolism, has been shown to have significant anti-inflammatory effects *in vitro* and vivo (25) and also have beneficial effects on the intestinal epithelial barrier function (26). Indolelactic acid, an upstream metabolite of indoleacrylic acid has been shown to possess antimicrobial, anti-oxidative, anti-inflammatory activities (26, 27) and can potentially modulate immune function (28). L-5-hydroxytryptophan (5-HTP) is a monoamine neurotransmitter involved in the modulation of mood, cognition, reward, learning, memory, sleep, and numerous other physiological processes (29), and can also suppress inflammation and arthritis by decreasing the production of pro-inflammatory mediators (30). Overall, MLs, especially FMLs, must endow anti-bacterial, anti-oxidant, anti-inflammation, and immunity-enhancing properties *via* tryptophan metabolism.

Linoleic acid (LA), arachidonic acid (ARA), eicosapentaenoic acid (EPA) and α-linolenic acid (ALA) are representative of the main PUFAs, and the major metabolic pathways of peroxidation described in mammals are both enzymatic (cyclooxygenase, COX; lipoxygenase, LOX; cytochrome P450, CYP) and non-enzymatic (31) oxidation. 8-HETE and 13-HOTrE are all oxylipins, a group of oxidized metabolites derived from PUFAs (32). Generally, the synthesis of oxylipins fluctuates with the changes of physiological or pathological states (33).13-HOTrE is derived from ALA *via* the COX enzymatic pathway. Studies have revealed that 13-HOTrE levels are significantly increased in some diseases (34, 35), such as acute liver injury. Therefore, it is generally thought to be a proinflammatory factor. HETEs are derived from ARA through COX catalysis. Hayashi et al. (36) reported that several ARA-derived (18-HETE/20-HETE) and ALA-derived (13-HOTrE) oxylipins tend to increase in bovine mastitic milk. In this study, MLs treatments reduced the 8-HETE contents. Meanwhile Ma et al. (37) also reported that 8-HETE is relevant for the efficacy of Zuojin pill treatment in chronic nonatrophic gastritis, as the level of 8-HETE was higher before treatment than after treatment. Thus, decreased oxylipins in this study with MLs treatments probably improve the antioxidant performance and immunity of the body and will be promising markers for livestock welfare.

8-iso Prostaglandin F2α, as a final product of lipid peroxidation, is generated from ARA interacting with ROS through nonenzymatic routes and is a robust oxidative stress biomarker of some diseases (32, 38). 8-iso Prostaglandin F2β is a constitutional isomer of 8-iso Prostaglandin F2α. Oliveira et al. (39) found that 8-iso Prostaglandin F2β has much lower potency than 8-iso Prostaglandin F2α with an α-configuration. 11β-Prostaglandin F2α, as a metabolite of 8-iso Prostaglandin F2α, have been found to be associated with levels of oxidative stress in specific diseases (40). Thus, the markedly decreased 8-iso Prostaglandin F2α, 11β-Prostaglandin F2α, and 8-iso prostaglandin F2β levels after MLs treatment indicate a decline in the peroxidation of PUFAs, which will produce benificial effects on lowering oxidative stress and enhancing disease resistance.

Carnitine plays a key role not only in fatty acid β-oxidation, but also in immunity enhancement and disease resistance. Guo et al. (41) found that carnitine C8:1 was significantly decreased the in non-alcoholic steatohepatitis group, and this could be profoundly reversed after luteolin treatment. Studies have reported decreased serum acyl-carnitine concentrations in patients with cancer (42). It can be hypothesized that increased carnitine C8:1 levels altered by MLs supplementation might accelerate mitochondrial β-oxidation (43) thereby enhancing immunity and disease resistance.

Previous studies have shown that increased *GCNT1*, *IFITM10*, *EXTL1*, *RILP*, *BBC3*, *RAB9A*, *MOB3B*, *SESN1*, *CDH4*, *MEIS1*, *RAB9A*, *NUDT7*, and *FMO2* are related to immune deficiency, autophagy inhibition, disease sensitivity and oxidative stress. Therefore, after treatment of MLs, the decreased peroxidation of PUFAs (the decreasing in peroxidation products) reduced the expression of the above genes, thus improving the immune and antioxidant properties. In addition, Shumar et al. (44) and Kerr et al. (45) suggest that decreased *NUDT7* may reduce the accumulation of peroxisome through regulating the β-oxidation of peroxisome fatty acids, thus improving the antioxidant performance; Ge et al. (46) found that the decrease of *NUDT7* enhanced the immune defense response; Taniguchi et al. (47) and Liu et al. (48) reported that *NUDT7* with low expression may up-regulate heme biosynthesis and contribute to meat-redness enrichment. Therefore, the addition of MLs can not only reduce the peroxidation of PUFAs, enhancing the antioxidant capacity and immunity of the body, but also improve meat redness.

Overall, oxidation resistance is closely related to the immune response. Our study proved that MLs supplementation is effective in promoting oxidation resistance and disease resistance, which is attributed to its function in reducing peroxidation of PUFAs and increasing tryptophan metabolism. Additionally, as lipid oxidation products affect the shelf life (49), sensory characteristics (50), and nutritional composition of meat (51), the role of MLs in reducing the peroxidation of PUFAs is speculated to be linked to the improvements in meat quality.

### Immune response

According to Sundling et al. (52), secreted antibodies confer immune protection by first attaching to foreign antigens through the paired variable regions of their immunoglobulin heavy and light chains. Immunity was enhanced with increased Ig, IgG and reduced TNFα in both MLs treatments. In addition, the FMLs induced maximum immunity in animals with a transition in antibody class from IgM to IgG, over the course of an immune response (14), During which, early low-affinity IgM antibodies are progressively replaced by more-effective, high-affinity IgG antibodies (53) to achieve effective serological immunity (52).

*DOCK2* regulates the migration of certain subsets of immune cells *via* Rac activation (54) and plays an important anti-inflammatory role in the development of various inflammatory diseases (55). *BBC3* is a transcriptional apoptotic target gene and participates in the activation of cell death processes (56). Pozo et al. (57) reported that pro-inflammatory *MYO10* mediates inflammation in cancer by regulating genomic stability. Studies have shown that *PIK3R3* is a multifunctional gene related to inflammatory diseases, livestock coat color, and cell proliferation (58–60). Shao et al. (61) clarified that *PLA2G4D*, a major pro-inflammatory factor, facilitates CD1a expression, which can be recognized by lipid-specific CD1a-reactive T cells, leading to the production of IL-22 and IL-17A. According to Ehmsen et al. (62) and Jiang et al. (63), the increased expression of *GADD45A*, a cell cycle regulator, can ameliorate liver fibrosis in rats and is a protective modifier of neurogenic skeletal muscle atrophy. Collectively, MLs supplementation improves muscle immune response and disease resistance.

Glycolysis is a critical process closely related to the immune response, as well as provides biosynthetic intermediates and reducing power for cell growth and proliferation of immune cells (64). The pentose phosphate pathway (PPP) from glucose-6-P to glyceraldehyde-3-P provides immune cells with key metabolites for immune function, such as reducing power for the synthesis of ROS and antioxidants in phagocytic cells and for phospholipid synthesis in dendritic cells. The hexosamine biosynthesis pathway, originating from fructose-6-P, provides substrates for the glycosylation of lipids and proteins that are important for Treg and M2 macrophage lineages (64). Thus FMLs treatment may enhance immunity by glycolysis, which in turn provides key metabolites for immune function.

D-mannose serves a vital function in T cell immune responses and is currently receiving increasing attention, although its normal physiological blood concentration is less than one-fiftieth of that of glucose. Zhang et al. (65) recognized that D-mannose induces regulatory T cells and suppresses immunopathology both *in vivo* and *in vitro*. Mannose-6-phosphate metabolized by D-mannose is a novel regulator of T cell immunity (66) and a promising target ligand in cancer therapy, as well as confers a better efficacy and lower toxicity in healthy tissues (67). Moreover, mannose-6-P not only plays a crucial role in lysosomal functions (such as autophagy) but also in regulating lysosome biogenesis (68). Thus, significantly increased D-mannose in DMLs treatment (*p*<0.05) and mannose-6-P in FMLs treatment (*p*<0.05) (**Figure 6**) *via* the mannose-6-P pathway enhances T cell immunity and likely regulates the lysosome biogenesis in autophagy.

Taken together, FMLs supplementation could improve the immune response *via* glycolysis and the mannose-6-P pathway and induce class switch from low-affinity IgM to high-affinity IgG antibodies.

## Data availability statement

The original contributions presented in the study are publicly available. This data can be found in the NCBI repository under accession number: PRJNA898816 [https://www.ncbi.nlm.nih.gov/search/all/?term=PRJNA898816] and in the MetaboLights repository under accession number: MTBLS6516.

## Ethics statement

The animal study was reviewed and approved by Animal Care and Use Committee of the Institute of Northwest A & F University. Written informed consent was obtained from the owners for the participation of their animals in this study.

## Author contributions

XC and YY contributed to the conception and design of the study. XC performed the statistical analysis and wrote the first draft of the manuscript. YY, MZ, FJ, LB, CS, and YQ revised the manuscript. SL, HW, ZL, XW, WQ and XS helped with the experimental sections. CS and YQ provided financial support for the manuscript. All authors contributed to manuscript revision, and read and approved the submitted version.
